# The experimental study of TNF-α & CRP expression in the spinal tuberculosis after instrumentation

**DOI:** 10.1016/j.amsu.2021.103048

**Published:** 2021-11-12

**Authors:** Tjuk Risantoso, Mohammad Hidayat, Hidayat Suyuti, Aulann'iam

**Affiliations:** a Medical Faculty of Brawijaya University, Saiful Anwar General Hospital, Malang, Indonesia; b Medical Faculty of Brawijaya University, Malang, Indonesia; c Opthalmology Department and Biochemistry Service Affiliation, Medical Faculty of Brawijaya University, Malang, Indonesia; d Faculty of Veterinary Medicine, Brawijaya University, Malang, Indonesia

**Keywords:** TNF-α, CRP, Spondylitis tuberculosa, Instrumentation, Inflammation

## Abstract

**Introduction:**

Previously, the management of spinal TB was using drugs and external stabilization. Surgical techniques were developed afterwards to clean the infected vertebral segment. The TB treatment approach is now based on immunology because the bacteria *Mycobacterium tuberculosis* has unique characteristics and the increasing cases of MDR (multiple drug resistant) TB due to mutation processes. TNF-α and CRP has a major role in immune activity of spinal TB. The energy from metal devices composed of ions and particles that have been used in instrumentation is expected to reduce the biomolecular and biocellular activity of the spinal tuberculosis inflammation activity. This study aims to investigate TNFα and CRP value as evaluator of bone inflammation activity in Spinal TB through experimental studies in Laboratory at Veterinary Faculty, Universitas Brawijaya.

**Methods:**

We investigates 40 New Zealand Rabbits which were given TB H37Rv strain infection in the vertebral body. Samples were divided into five groups namely control rabbits, infected rabbits without intervention, infected rabbits treated by instrumentation, infected rabbits given anti-tuberculosis drugs and infected rabbits treated by instrumentation and given drugs. The cytokine levels of TNF-α and CRP were evaluated and compared as the main outcome.

**Result:**

The results showed a notable TNF-α and CRP decrease in infected rabbits given drugs alone and instrumentation alone compared to infected rabbits without intervention. There was a significant TNF-α and CRP decrease in infected rabbits given drugs and treated by instrumentation compared to control rabbits and rabbits who received drugs only.

**Conclusion:**

Instrumentation can reduce the inflammation activity in spinal tuberculosis by affecting the body's cytokine levels.

## Highlights

• In conclusion, the use of metal/titanium instruments to produce energy emissions through the instrument directly affect the immune activity of bone inflammation.
• Limitation of this study that this research is still using experimental animals.
• Further research should explain clearer information about the implant energy resonance activity in diseased tissues.

## 1 Introduction

*Mycobacterium tuberculosis* infection occurs when several popular airborne tubercle bacilli from a patient with pulmonary TB reach the alveoli of the host. Here, Mtb is rapidly phagocytosed by alveolar macrophages which can normally kill bacteria that enter by innate immune response (Innate Immune Response) [1]. If the bacillus can survive this first line of defense, it begins to actively replicate in macrophages [2]. During the early stages of this infection, Mtb can diffuse to other organs via lymphatics and through hematogenous spread where it can infect other cells including the spine [2]. After that, once the adaptive immune response appears, neutrophils, lymphocytes, and other immune cells to the primary infection site form cellular infiltrates which then form a typical granuloma structure [[3], [4], [5]].

The clinical manifestations of *Mycobacterium tuberculosis* infection describe the complex interactions between the causative bacteria and the host immune response. The immune response to TB infection includes innate and adaptive immune responses. The innate immune response is played by macrophage cells, while the adaptive immune response is played by T lymphocytes, both CD4 +, CD8 + [6,7]. TNF-α is referred to as multifunctional cytokines, proinflammatory cytokines and is produced by dendrites, macrophages, and T cells. These cytokines have a role in the recruitment of phagocytosis cells by stimulating or activating other macrophage/dendrite cells. Besides that, it stimulates and activates Th1 cells by stimulating IFN-. IL-6 is a pro-inflammatory and inflammatory cytokine produced by macrophages. IL-6 has activities in collaboration with TNF-α to continue advanced stage inflammation. IFN-γ, is a cytokine needed by adaptive immunity cells, namely CD4-Th1 cells, in regulating NK cells - as a direct killer of Mycobacterium. IL-17 is a cytokine produced by Th-17 cells and has the ability to reactivate the adaptive immunity response to Mycobacterium and is useful for delivering the healing activity of Spondylitis tuberculosa [8].

Immune cells and macrophages are the first cells to interact with biological materials. Macrophages are responsible for cleaning wounds, inflammation, and recruiting tissue-producing cells. Macrophages are activated as pro-inflammatory (M1) or anti-inflammatory (M2); activation of macrophages will control the inflammatory response environment. Macrophage activation is generally characterized by pro-inflammatory and anti-inflammatory cytokine and chemokine cells. The purpose of this study is to describe the effects of microstructure and surface energy on cytokine production and macrophage activation. The M2 anti-inflammatory response is manifested by high levels of IL4, IL10, and TGFβ [[9], [10], [11], [12], [13]]. According to the data, the modification of the surface changes the response of the immune system and the cytokines released by the cells [12,13]. This leads to a recruitment of cells capable of regenerating tissue rather than chronic inflammation and fiber encapsulation. Macrophage activation can be induced or regulated by the characteristics of the implant surface, which will eventually change the healing activity and affect the long-term stability of the implant. A chronic immune response prevents the formation of healthy tissue around the implant [[14], [15], [16]]. This study aimed to investigate the expression of TNFα and CRP in Tuberculous Spondylitis (TB) and its relationship with instrumentation.

This study used New Zealand white rabbits as samples to study the expression of TNFα and CRP as a marker of the bone inflammation activity of spondylitis. The experimental study used experimental animals that allowed the control of variables. Several studies have shown that New Zealand white rabbits can use the aerosol infection system to infect *Mycobacterium tuberculosis* bacteria. After 633 weeks of exposure to laboratory strains of *Mycobacterium tuberculosis* (H37Rv), they will develop spinal cord infections [16].

## 2 Methods

Like a former study, this study adopts a purely in vivo experimental design, randomized post-test only a control group design, 45-month-old New Zealand male rabbits (*Oryctolagus cuniculus*) weighing 3000 g as the sample. The rabbits were obtained from PT Bio Farma (Persero) Bandung. During the research process, rabbits were treated in a special environment free of pathogens in accordance with the recommendations of the Federation of European Laboratoy Animal Science Associations (FELASA) which had been approved and received a certificate of ethical feasibility from the Animal Care and Use Committee Brawijaya University. The tuberculous spondylitis model was developed by infecting the H37Rv strain of *Mycobacterium tuberculosis* in the spine. Because the total population of Rabbits in the world is unknown, the sample size is calculated based on the Federrer replication formula. The samples are divided into 5 groups and each group consist 8 rabbits. The inclusion criteria of the sample are healthy New Zealand male white rabbit, weighs 3000–3500 g, aged between 12 and 16 weeks, skeletally mature and do not have spinal deformities due to trauma or congenital abnormalities. And the exclusion criteria are rabbits that declared by a consultant veterinarian to be diseased within a period of clinical evaluation under appropriate environmental conditions (for 7 × 24 h) and failure to achieve the 4 week bacterial incubation period (rabbits die). The first group is normal rabbits. The second (K+) was only infected with tuberculosis infection (*Mycobacterium tuberculosis*) strain H37Rv. The third (KOAT) consisted of rabbits infected with TB (*Mycobacterium tuberculosis*) strain H37Rv, followed by a single oral antituberculous drug (OAT), namely rifampicin. The fourth (KOAT) consisted of rabbits with TB infection (*Mycobacterium tuberculosis*) strain H37Rv, followed by titanium kits. The fifth (KI + OAT) used rabbits to treat tuberculosis infection (*Mycobacterium tuberculosis*) strain H37Rv, followed by operation of the device and combined oral anti-tuberculosis drugs. The CRP and TNFα level was measured 2 weeks after instrument and drug administration [16]. The whole experiment was monitored and controlled in each phase.

The rabbits were anesthetized with intraperitoneal xyla (25 mg kg) and ketamine (50 mg/kg). A biopsy of the spine is performed by cutting a 50 mm cross section of the spine. Biopsy results are stored in a 2 ml test tube containing 10% formalin solution for histological and immunohistochemical preparation [16].

### 2.1 Histochemical preparation

For analysis and histopathological examination, the tissue is processed for preparation. The following describes the paraffin block preparation procedure: Tissues are dehydrated for 60 min each with multigrade alcohol (30%, 50%, 70%, 80%, 96% and anhydrous). Purge with xylene, twice each time, 60 min each time. The next day, the paraffin block was fixed on the support and cut 46um thick with a rotary microtome. From each section of the paraffin block, one preparation was stained with hematoxylin, while the other preparation was used for immunohistochemical staining.

### 2.2 Hematoxilen-eosin staining

The slides were washed with PBS pH 7.4 for 5 min, and then stained with threo-xylene for 10 min. Afterwards, soak the slides in tap water for 10 min and rinse with dH2O. The next step is to dehydrate with 30% and 50% alcohol for 5 min, and then dye with eosin solution for 3 min. Then the slides were rinsed with 30% alcohol, washed with dH2O for 5 min, and then air-dried. The last step is the entangled installation and cover glass coating [16].

### 2.3 Immunoperosidase towards TNFα and mt38

The formulation was dewaxed with xylene for 15 min and then rehydrated with 100% and 70% alcohol for 10 min. They were washed twice with dH2O and incubated with PBS solution for 5 min. The preparation is stored in a glass box containing citrate buffer and autoclaved for 15 min to optimize its antigenicity. They are cooled to room temperature for an hour and after a short drying period, lines are drawn on the paper towels with a pen and paper to separate the paper towels. Before incubating with 0.3% H 2 O 2 for 15 min, the formulation was washed with dH 2 O for 5 min, washed with PBS for 5 min and rinsed with PBS pH 7.2 3 times for 5 min each. Incubate with blocking solution for 30 min. Incubate with mouse monoclonal TNFα: SantaCruz cat or mouse monoclonal mt38 at 40 °C overnight. Rinse with PBS pH 7.4 and incubate on the secondary antibody. HRP was labeled with anti-mouse IgG for 1 h. They were washed with PBS pH 7.4 and incubated in the HRP substance DAB for 5 min. Wash with PBS pH 7.4, then wash with PBS pH 7.4. Re-stain with Mayer Hematoxylene for 10 min. Rinse them with dH2O. Drain and cover with a coverslip [16].

The data then analyzed the smallest significant difference (ANOVA) using SPSS ver.16. All statistical calculations were performed using the SPSS-16.0 computer program for Windows (SPSS Inc., Chicago, IL, USA). The data was tested using One-way Annova Test and Post hoc Tukey HSD Test. The difference was declared significant if the p value < 0.05.

## 3 Results

The study used the technique of infecting the tuberculosis strain H37Rv by injecting 100 cfu (colony forming units) into the spinal column (L4) of rabbits. Rabbits are the sample for the size of their spinal tissue and its relationship with the instrument, as well as for their response and resistance to tuberculosis infection.

In the histopathological examination, it was determined that the tissue lesions of the vertebral body were a tissue reaction to the bacterium *Mycobacterium tuberculosis*. After 400x magnification, a high monocyte distribution was found in the granuloma area. There is also the area of the granuloma stained with anti-mt38 immunohistochemically. Magnified 1000 times, a positive mt38 result is indicated by monocyte darkening. Both histopathological and immunohistochemical examinations showed good results in the evaluation of the infection, which shows that there were notable differences between all the experimental groups and the control groups.

**Fig. 1:**
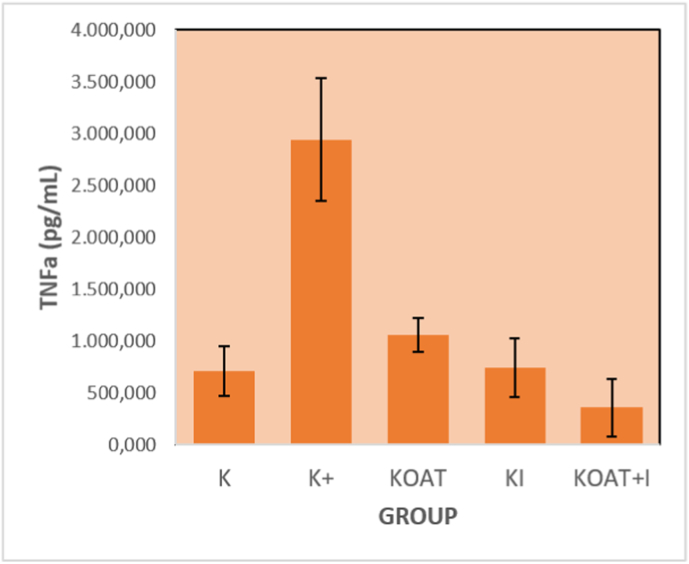
TNF-α Expressions based on the Experimental Groups.

Our research showed a notable decrease of TNF-α expression in the spine of TB spondylitis after the administration of OAT drug as shown in (Table 1) and (Fig. 1). TNF-α also decreased significantly after the instrumentation. The combination between instrumentation and OAT drug resulted in notable increase of TNF-α expressions compared to the control or the OAT drug only. Therefore, the instrumentation had a major role towards TNF-α expressions in the TB spondylitis model in relation to inflammation activity of the bone.

**Table 1:** TNF-α Expressions based on the Experimental Groups.

GROUP	AVERAGE (pg/ml)	+SD	p-value
**K**	708.4167	242.88746	0.000*
**K+**	2938.0667	593.70993	
**KOAT**	1055.0833	162.63261	
**KI**	742.5000	279.82472	
**KOAT + I**	357.4167	278.76974	

*One-way Annova Test. Post hoc Tukey HSD Test: K+ vs KOAT: p = 0.047; K+ vs KI: p = 0.0001; K+ vs KOAT + I: p = 0.0001; KI vs KOAT + I: p = 0.000.

K: K+: infected group without treatment; KOAT: infected group treated with oral anti tuberculosis drugs only; KI: infected group treated with instrumentation only; KOAT + I: infected group treated with instrumentation and oral anti tuberculosis drug.

**Fig. 2:**
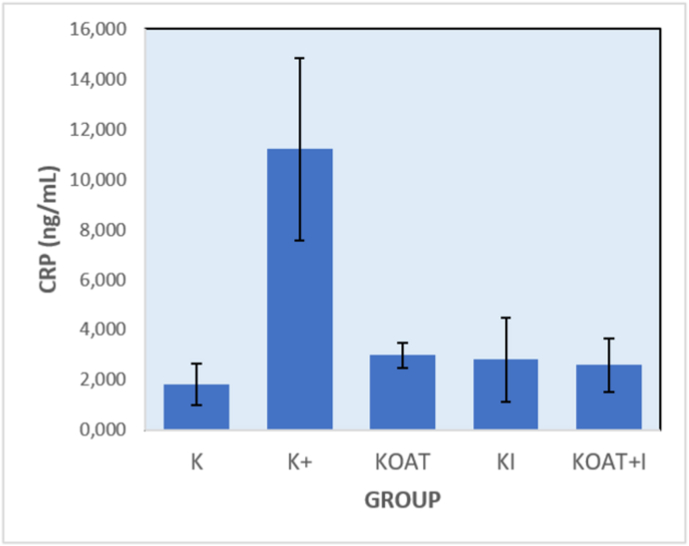
CRP Expressions based on the Experimental Groups.

Findings also showed a notable decrease of CRP expression after the administration of OAT drug and also after instrumentation procedure as shown in (Table 2) and (Fig. 2). Between the OAT drug and instrumentation, there was no notable difference, but combination between the two resulted in notable increase of CRP compared to the control or the OAT drug only. Therefore, the instrumentation also had a major role towards CRP expressions in the TB spondylitis model in relation to inflammation activity of the bone.

**Table 2:** CRP Expressions based on the Experimental Groups.

GROUP	AVERAGE (ng/ml)	+SD	p-value
K	1.817	0.8353	0.000*
K+	11.200	3.6277	
KOAT	2.983	0.5115	
KI	2.817	1.6762	
KOAT + I	2.600	1.0640	

*One-way Annova Test. Post hoc Tukey HSD Test: K+ vs KOAT: p = 0.047; K+ vs KI: p = 0.0001; K+ vs KOAT + I: p = 0.0001; KI vs KOAT + I: p = 0.000.

K+: infected group without treatment; KOAT: infected group treated with oral anti tuberculosis drugs only; KI: infected group treated with instrumentation only; KOAT + I: infected group treated with instrumentation and oral anti tuberculosis drug.

## 4 Discussion

### 4.1 Tb spondylitis in the rabbit

Like the former study, the inoculation of the bacterium *Mycobacterium tuberculosis* in the vertebral body was carried out by making defects or drilling holes. Histopathological examination showed that vaccination was successful, because each examination result was positive. Successful bacterial inoculation depends on several factors, namely the host (rabbit), bacteria, and the environment. The host (rabbit) plays a key role in managing this procedure (bacterial inoculation) [16].

Technical factors are another important factor for bacterial inoculation. The vaccination procedure performed in this study is similar to the vaccination procedure of the previous study, which is a model of osteomyelitis in the host bone. One problem the researchers found was related to the type of bacteria used in the inoculation process. You have never been vaccinated against *Mycobacterium tuberculosis* before [16].

Another aspect that affects inoculation is the type and preparation of *Mycobacterium tuberculosis* bacteria. Because the bacteria are obtained from the laboratory, their virulence is expected to be different from that of bacterial strains obtained directly from the environment. The concentration of bacteria is another important factor that was studied in this study [16].

The concentration of *Mycobacterium tuberculosis* used in this study was between 10^6^ cfu/ml and 10^8^ cfu/ml. In general, 10^4^ - 10^6^ organisms/ml is a requirement for TB testing and is the basis for determining the minimum concentration used in this study.

### 4.2 Inflammatory cytokines in Tb spondylitis

There are many unique processes and actions regarding host – agent interactions, especially regarding the immunological activity against TB which is controlled by the human immune system known as Mediated Immune Response (cell defense and cell products such as cytokines and growth factors). The archestral of the inflammatory activity and followed by the healing activity is a complex and comprehensive route.

The literature states that TNF-α, IFN-γ, IL-6, CRP and IL-7 are the cytokines that most often contribute significantly to TB infection. The activity of these cytokines starts from the invasion and incubation of TB microbes to systemic spread/bacteremia. The microbial killing activity will be followed by the regulation and coordination of cytokines, especially TNF-α, IFN-γ, IL-6, CRP and IL-7. There are many types of processes to kill bacteria such as: a/direct killing by NK cells, b/apoptosis with granuloma formation, c/intra-cellular phagocytosis. All these processes are regulated by immunological defense processes and carried out by a mediated immune response, that is, cell defense with molecular products [[16], [17], [18], [19], [20]].

## 5 Conclusion

In conclusion, the use of metal/titanium instruments to produce energy emissions through the instrument leads to a decrease in TNFα and CRP, which directly affect the immune activity of bone inflammation. Limitation of this study that this research is still using experimental animals, not yet on humans and the use of materials in this study only uses 1 type of material. Further research should explain clearer information about the implant energy resonance activity in diseased tissues, which activates the immune activity to kill bacteria and reduce the inflammation activity.

## Author contribution

Tjuk Risantoso: Study Concept, Data Collection, Data analysis, Writing the paper. Mohammad Hidayat: Study Concept. Hidayat Suyuti: Study Concept. Aulanni'am: Study Concept.

## Financial disclosure

Tjuk Risantoso, Mohammad Hidayat, Hidayat Suyuti and Aulani Niam have not received research grants from any sources.

## Human rights

This article does not contain any studies with human subjects performed by the any of the authors.

## Animal studies

All institutional and national guidelines for the care and use of laboratory animals were followed. The animals were obtained from PT Bio Farma (Persero) Bandung. During the research process, animals were treated in a special environment free of pathogens in accordance with the recommendations of the Federation of European Laboratoy Animal Science Associations (FELASA) which had been approved and received a certificate of ethical feasibility from the Animal Care and Use Committee Brawijaya University.

## Ethical approval

All institutional and national guidelines for the care and use of laboratory animals were followed.

## Please state any sources of funding for your research

Tjuk Risantoso, Mohammad Hidayat, Hidayat Suyuti and Aulani Niam have not received research grants from any sources.

## Please state any conflicts of interest

Tjuk Risantoso, Mohammad Hidayat, Hidayat Suyuti and Aulani Niam declare that we have no conflict of interest.

## Registration of research studies

1. Name of the registry:
2. Unique Identifying number or registration ID:
3. Hyperlink to your specific registration (must be publicly accessible and will be checked):

## Guarantor

Tjuk Risantoso.

## Consent

This article does not contain any studies with human subjects performed by the any of the authors.

## Annals of medicine and surgery

The following information is required for submission. Please note that failure to respond to these questions/statements will mean your submission will be returned. If you have nothing to declare in any of these categories then this should be stated.

## Declaration of competing interest

Tjuk Risantoso, Mohammad Hidayat, Hidayat Suyuti and Aulani Niam declare that we have no conflict of interest.
